# C-Reactive Protein Promotes the Activation of Fibroblast-Like Synoviocytes From Patients With Rheumatoid Arthritis

**DOI:** 10.3389/fimmu.2020.00958

**Published:** 2020-05-20

**Authors:** Zhengyu Fang, Jiyang Lv, Jing Wang, Qingxia Qin, Juan He, Meiying Wang, Gengmin Zhou, Guoyu Liu, Fubo Zhong, Yadan Zheng, Hui-Yao Lan, Qingwen Wang

**Affiliations:** ^1^Department of Rheumatism and Immunology, Peking University Shenzhen Hospital, Shenzhen, China; ^2^Biomedical Research Institute, Shenzhen Peking University - The Hong Kong University of Science and Technology Medical Center, Shenzhen, China; ^3^Department of Medicine and Therapeutics, Li Ka Shing Institute of Health Sciences, and Lui Che Woo Institute of Innovative Medicine, The Chinese University of Hong Kong, Hong Kong, China

**Keywords:** RA, FLS, CRP, CD32, CD64, p38, NF-κB

## Abstract

**Objective:** To evaluate the biological effect and mechanisms of C-reactive protein (CRP) on the activation of fibroblast-like synoviocytes (FLSs) from patients with rheumatoid arthritis (RA).

**Study design:** To understand if CRP is involved in RA, expression of CRP and its receptors CD32/64 was examined in synovial tissues from RA patients and normal controls. *In vitro*, the potential role and mechanisms of CRP in FLS proliferation and invasion, expression of pro-inflammatory cytokines, and activation of signaling pathways were investigated in both RA - FLS and a normal human fibroblast-like synoviocyte line (HFLS).

**Results:** Compared to normal controls, synovial tissues from 21 RA patients exhibited highly activated CRP signaling, particularly by FLSs as identified by 65% of CRP-expressing cells being CRP+vimentin+ and CD32/64+vimentin+ cells. *In vitro*, FLSs from RA patients, but not HFLS, showed highly reactive to CRP by largely increasing proliferative and invasive activities and expressing pro-inflammatory cytokines and chemokines, including CCL2, CXCL8, IL-6, and MMP2/9. All these changes were blocked largely by a neutralizing antibody to CD32 and, to a less extent by the anti-CD64 antibody, revealing CD32 as a primary mechanism of CRP signaling during synovial inflammation. Further studies revealed that CRP also induced synovial inflammation differentially via CD32/CD64- NF-κB or p38 pathways as blockade of CRP-CD32-NF-κB signaling inhibited CXCL8, CCL2, IL-6, whereas CRP induced RA-FLS invasiveness through CD32-p38 and MMP9 expression via the CD64-p38-dependent mechanism.

**Conclusions:** CRP signaling is highly activated in synovial FLSs from patients with RA. CRP can induce synovial inflammation via mechanisms associated with activation of CD32/64-p38 and NF-κB signaling.

## Introduction

Rheumatoid arthritis (RA) is a chronic inflammatory disease characterized by polyarticular and symmetrical arthritis mainly affecting the facet joints and is a major cause leading to progressive joint destruction and functional impairment or disability. RA is also an autoimmune disease, though pathogenic mechanisms remain to be clarified (1, 2).

Activated FLSs in the invasive pannus tissue have been shown to play a major role in the crippling destruction of joint cartilage and bone in patients with RA (3, 4). Several studies indicate that RA-derived FLSs are morphologically altered and expressed major histocompatibility complex (MHC)-II, pro-inflammatory cytokines, adhesion molecules, proangiogenic factors, and matrix degrading enzymes (5, 6). Activated RA-FLSs also cause erosive arthritis with alterations in a variety of inflammatory genes and signaling cascades (7–11).

Elevated serum levels of C-reactive protein (CRP) have been shown to correlate with the disease activity in patients with RA (12). CRP is secreted by the liver in response to a variety of inflammatory cytokines. In patients with RA, increased levels of CRP are also found in the synovial fluid (13, 14), although the source origin of CRP remains unclear. It is well-known that the interaction of CRP with Fc-γ receptors leads to the production of pro-inflammatory cytokines (15, 16). Here we tested the hypothesis that CRP may be produced locally by FLSs and functions to induce the synovial inflammation in patients with RA.

## Materials and Methods

### Patients

Synovial tissues were obtained from a total of 21 patients fulfilling the American College of Rheumatology classification criteria for RA. A list of the detailed clinical history of the enrolled RA patients was shown in Table 1. In addition, 3 normal human control synovial tissues (HNC) were obtained from surgically removed joint tissues from individuals who suffered from traumatic joint damage. All patients were informed about the aims of specimen collection and given signed written consent. The study was approved by the Human Research Ethics Committee and performed in accordance with the ethical guidelines of Peking University Shenzhen Hospital.

**Table 1:** Clinical characteristic of the RA patients (*n* = 21) enrolled in the present study.

**Characteristic**	**Value**
Female gender, *n* (%)	85.7
Age (years)	50.1 ± 8.1
Weight (kg)	62.8 ± 10.3
BMI (kg/m2)	28.7 ± 4.9
Disease duration (years)	7.8 ± 4.9
Duration of morning stiffness (h)	2.8 ± 1.9
Tender joint count	10.3 ± 5.9
Swollen joint count	5.5 ± 3.1
ESR (mm/1st hr)	38.5 ± 15.1
CRP (mg/l)	21.8 ± 13.8
Anti-CCP titer (UI/ml)	136.2 ± 31.8
RF titer (UI/ml)	157.8 ± 29.9
Anti CCP positivity, *n* (%)	80.1
RF positivity, *n* (%)	82.8
VAS (0–100)	59.8 ± 10.3
DAS28-CRP	4.98 ± 0.66
DAS28-ESR	5.29 ± 0.71

### Primary Culture of FLSs

For *in vitro* studies, synovial FLSs were isolated from the biopsied synovial tissues of 4 RA patients and cultured in Dulbecco's modified Eagle medium (DMEM, Thermo Fisher Scientific, Waltham, USA) containing 10% FCS as described previously (17). Human fibroblast-like synoviocytes (HFLS, Cell Applications, Inc., San Diego, CA) were used as normal control. A human recombinant CRP used in this study is homo-pentameric (26 kDa) with predicted molecular mass (monomer) at 23 kDa (R&D Systems, Minneapolis, MN). Both RA-FLSs and HFLSs were stimulated with a CRP (10 μg/ml) in the presence or absence of neutralizing antibodies CD32/64 (10 mg/ml, R&D Systems) for hours 0, 3, 6, 12, and 24. mRNA expression of pro-inflammatory cytokines and chemokines including IL-1β, IL-6, CXCL8, IL-10, CCL2, and MMP9 were detected by real-time PCR and protein levels were measured by multiplex cytokine assay kits (Bio-Rad, Hercules, CA, USA) according to the instructions of the manufacturer. For cell proliferation assay, FLSs and HFLSs were cultured in 96-well culture plates (1 × 103 per well) for days 0, 1,2,3,4, and 5 with or without CRP (10 μg /ml) and the cell proliferating activity was determined by the WST-1 assay following the manufacturer's instructions (Roche, Basel, Switzerland).

We also examined the effect of CRP on FLS invasive activities by the transwell migration assay. Briefly, after treated with CRP (10 μg /ml) in the presence or absence of neutralizing antibodies CD32 or CD64 (10 mg/ml) for overnight, 200 μL of FLS suspension containing niclosamide (Biovision, ABIN629143, Milpitas, CA, USA) was added to the upper compartments, while DMEM/F12 containing 15% FBS was placed in the lower chamber for 16 h at 37°C under 5% CO_2_. After incubation, the non-migrating cells were removed from the upper surface of the filter using a cotton swab. The filters were fixed in methanol for 15 min and stained with 0.1 % crystal violet (Santa Cruz Biotechnology, sc-214780A, CA, USA) for 15 min. Migration was quantitated by counting the stained cells that migrated to the lower side of the membrane using an optical microscope (1000x magnification). All experiments were performed in duplicate and repeated for at least 3 independent experiments.

To examine whether CRP induces NF-κB nuclear translation, immunofluorescence and subcellular fractionation were performed. First, 1.5 × 10^4^ RA-FLSs were seeded per well in a 4- chamber slide and then stimulated with or without CRP (10 μg /ml) for 12 h for immunofluorescent staining with a mouse monoclonal antibody against NF-κB/p65 subunit as described below. For subcellular fractionation(nuclear vs. cytoplasmic location) of NF-κB/p65 subunit, RA-FLSs were cultured with CRP (10 μg /ml) in the presence or absence of an neutralizing antibody CD32 (10 mg/ml) and subjected to western blot analysis with an antibody to p65 subunit (Cell Signaling Danvers, MA) as previously described (18).

To further investigate the mechanisms through which CRP differentially regulates RA- FLS proliferation, invasion, and proinflammatory cytokine expression, we pretreated RA- FLSs with the inhibitors to NF-κB (PDTC 100 μM, Sigma-Aldrich, US) or p38 (SB202190 100 μM, Sigma-Aldrich, US) for overnight before CRP (10 μg /ml) stimulation.

### Immunohistochemistry

Synovial tissues from 21 patients with RA or from 3 normal control (HNC) were either fixed in formalin for immunoperoxidase staining with the antibodies to human CRP, CD32, and CD64 (R&D Systems) on paraffin- tissue sections (4 μm) or snap-frozen for two-color immunofluorescence with antibodies to vimentin, CRP, CD32, or CD64 (R&D Systems). Tissue sections stained with a non-specific isotype antibody (IgG1) were used as negative controls following the same immunostaining protocol. After washing in PBS, sections were counterstained with DAPI and examined under Olympus biological fluorescence microscope (IX2-ILL100).

For *in-vitro* immunofluorescent staining, RA-FLSs cultured in a 4-chamber slide were harvested and washed with PBS, the cells were then fixed in 4% paraformaldehyde (pH 7.4) for 10 min at 37°C and permeabilized by 0.1% Triton X-100. Then the cells were stained with a mouse monoclonal antibody against the p65 NF-κB or a non-specific isotype control IgG1 (Cell Signaling) at room temperature for 3 h, followed by a fluorescent dye–labeled secondary antibody along with nucleus (DAPI) for 45 min at room temperature at dark. After washing, the coverslip slide was air-dried and mounted with the mounting medium (containing antifade agent) and examined under Olympus biological fluorescence microscope (IX2-ILL100).

### RNA Extraction and Real-Time PCR Analysis

Total RNA was isolated from tissues by using AxyPrepTM Blood Total RNA MiniPrep Kit (Axygen, Tewksbury, MA, USA) according to the manufacturer's instruction. Quantitative PCR was performed in Bio-Rad Chromo4 real-time PCR system (Bio-rad, Hercules, CA, USA) with primers as listed in Table 2. The levels of targeted genes were expressed as the ratio relative to the β-actin mRNA in each sample.

**Table 2:** Primers used for qRT-PCR.

**Gene**	**Forward**	**Reverse**
CD32	GTGCCTCAGCCGTTCTTG	AACCTGTAACATAAGCGTTTCTCA
CD64	GGTCAGCGTGTTCCAAGAG	CACCTGTATTCACCACTGTCATT
IL-1β	GCTTATTACAGTGGCAATGAGGAT	TAGTGGTGGTCGGAGATTCG
IL-6	GCCACTCACCTCTTCAGAAC	GCAAGTCTCCTCATTGAATCCA
CXCL8	AGGACAAGAGCCAGGAAGAA	GGGTGGAAAGGTTTGGAGTATG
TNF-α	AGCAAGGACAGCAGAGGA	GGTCAGTATGTGAGAGGAAGAGA
CCL2	CTGTGCCTGCTGCTCATAG	CTTGCTGCTGGTGATTCTTCT
MMP-3	CAGCAAGGCATAGAGACAACAT	CGCACAGCAACAGTAGGATT
MMP-9	GATCCAAAACTACTCGGAAGACTTG	GAAGGCGGGGGCAAA
MMP-2	GGCCCCACAGGAGGAGAA	GGTGCTGGCTGAGTAGATCCA
ACTB	GACCTGACTGACTACCTCATGAAGAT	GTCACACTTCATGATGGAGTTGAAGG

### Western Blot Analysis

Protein samples were extracted using RIPA lysis buffer containing protease inhibitors and phosphatase inhibitors (Thermo Fisher Scientific, Waltham, MA, USA). 20–40 μg of the cell lysate, which were determined by the BCA protein assay, were separated on 12% SDS-PAGE gels and electrophoretically transferred onto nitrocellulose membranes (Bio-Rad, Hercules, CA, USA). After blocking, the membranes were incubated overnight at 4°C with primary antibodies to CD32, CD64 (R&D Systems, Minneapolis, MN,USA), p38, phospho-p38(Cell Signaling, US), IκBα, phospho-IκBα, phospho-IKK, phospho-p65, p65, and TWIST (Cell Signaling Technology, Danvers, MA,USA), and house-keeping protein controls such as β-actin, α-tubulin or laminin B1(BD Pharmingen, San Diego, CA, USA). After washing, the membranes were incubated with HRP-conjugated secondary antibodies (R&D Systems) for 1 h and detected by enhanced chemiluminescence (ECL, Amersham Pharmacia Biotech, USA) reaction. Positive immunoreactive bands were quantified and normalized by β-actin using κκ The Image G program (NIH).

### Statistical Analysis

Data obtained from this study are expressed as the mean ± SEM. The statistical analysis was conducted using SPSS software (SPSS, Inc., Chicago, IL, USA). The statistical significance of the differences among groups was tested using one-way analysis of variance or Student's *t*-test. Error bars are indicative of standard deviation. *p* < 0.05 or *p* < 0.01 was considered significant.

## Results

### CRP Is Produced Locally by FLSs in Synovial Tissues From Patients With RA

**Figure 1:**
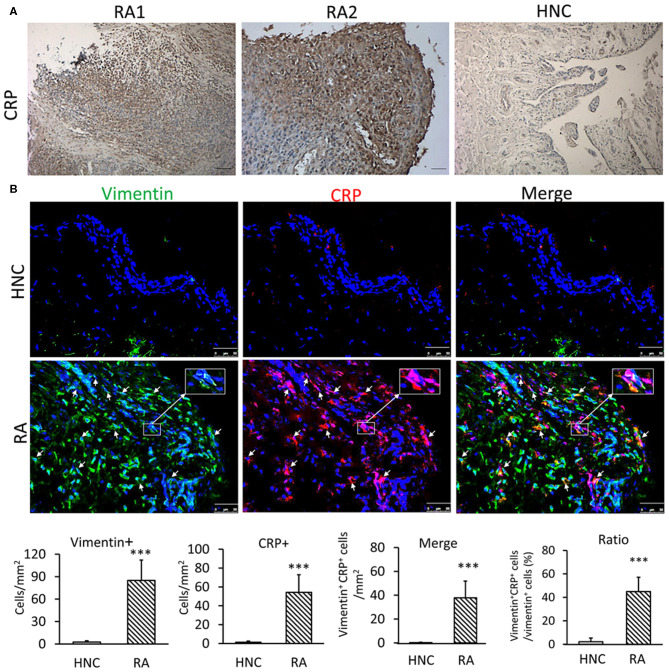
CRP is highly expressed by FLS in synovial tissues from patients with RA. **(A)** Representative immunohistochemical staining of CRP expression in synovial tissues from RA patients and healthy normal controls (HNC); A total of 21 RA samples and 3 HNC samples were included **(B)** Two-color immunofluorescence. Showing major CRP-expressing cells being FLSs by co-expressing CRP (red) and vimentin (green) antigens in synovial tissues of RA patients but not in HNC. Nuclei were stained with DAPI (blue). The inserted images are the enlargement showing vimentin+ CRP+ FLS from the indicated area. Vimentin+, CRP+, and vimentin+ CRP+ cells were accounted and expressed as cell/mm^2^ or percent CRP-expressing FLS (vimentin+ CRP+). Each bar represents the mean ± SEM for group of 21 RA patients and 3 HNC. ***p < 0.001 vs. control. Scale bar =50 μm.

It is well-known that synovial fluid of RA patients has high concentration of CRP (14). In the present study, we found that CRP was highly expressed in the synovial tissues of RA patients but not in normal controls (Figure 1A). Two-color immunofluorescence demonstrated more than 65% of CRP+ cells were FLSs identified by co-expressing vimentin in synovial tissues of patients with RA (Figure 1B). In contrast, no CRP-expressing cells were detectable in synovial tissues from normal controls (Figure 1B). This novel finding indicated that CRP was indeed produced locally in the synovial tissues of RA, particularly by FLSs.

### CRP Receptors CD32/CD64 Are Highly Upregulated in RA-FLSs From Synovial Tissues of RA Patients

**Figure 2:**
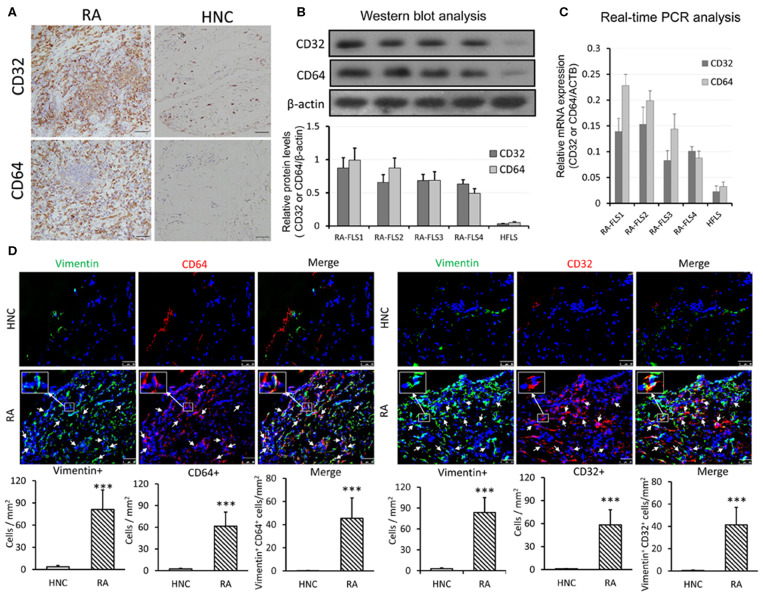
CRP receptors CD32/CD64 are highly upregulated in RA-FLSs from synovial tissues of RA patients. **(A)** Representative immunohistochemical staining of synovial tissues from RA patients(*n* = 21) and healthy normal controls (HNC, *n* = 3) with anti-human CD32 and CD64 antibodies; **(B)** Western blot analysis of CD32 and CD64 expression by RA-FLS and HFLSs. **(C)** Real-time PCR analysis of CD32 and CD64 mRNA expression by RA-FLS and HFLS. **(D)** Two-colorimmunofluorescence detects CD32 and CD64-expressing FLS (CD32+ or CD64+ vimentin+, arrowheads) in RA or HNC synovial tissues using anti-human vimentin, CD32 and CD64 monoclonal antibodies. Nuclei are stained with DAPI (blue). The inserted images are the enlargement showing CD32+vimentin+ and CD64+vimentin+ cells from the indicated area. Vimentin+, CRP+, and vimentin+ CRP+ cells were accounted and expressed as cell/mm^2^. Each bar represents the mean ± SEM for group of 21 RA patients and 3 HNC. ***p < 0.001 vs. control. Scale bar = 50 μm.

We next examined if CRP signaling is activated locally in the inflamed synovial tissues in patients with RA by examining expression of its receptors, Fcγ receptor I (CD64) &IIa (CD32). As shown in Figure 2A, immunohistochemistry revealed that most synovial cells from RA patients highly expressed both CD32 and CD64. In high contrast, only a few cells expressing CD32/64 were observed in the normal control synovial tissues. Similar results were also detected at the protein levels by western blot and at the mRNA level by real-time PCR (Figures 2B,C). Further study by using two-color immunofluorescence clearly identified about 65–70% of CD32/64-positive cells were FLS as demonstrated by co-expressing vimentin (Figure 2D). These findings suggest that CRP signaling is highly activated in FLS of patients with RA and may play a key role in the activation of inflammatory FLS during RA.

### CRP Promotes RA-FLS to Produce Pro-inflammatory Cytokines and to Become Highly Proliferative and Invasive Phenotype *in vitro*

**Figure 3:**
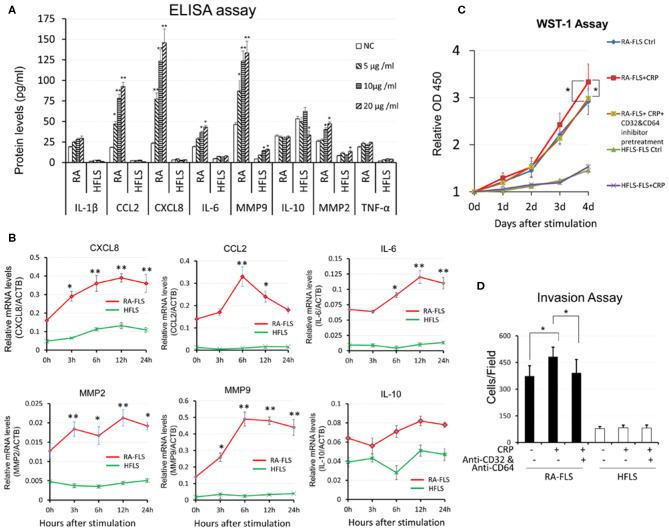
CRP promotes pro-inflammatory responses in RA-FLS. **(A)** CRP dose- dependently promotes RA-FLSs but not HFLSs production of pro-inflammatory cytokines including CCL2, CXCL8, IL-6, MMP9, MMP2 without altering IL-1β and TNFα levels as determined at the protein levels by ELISA. **(B)** Real-time PCR shows that CRP (10 μg/ml) significantly upregulates mRNA levels of CCL2, CXCL8, IL-6, MMP9, MMP2 at time-dependent manner. Interestingly, addition of CRP reduces IL-10 expression. **(C)** WST-1 assay shows that addition of CRP (10 μg/ml) promotes RA-FLS but not HFLS proliferation. **(D)** Invasion assay reveals that addition of CRP (10 μg/ml) for 24 h promotes RA-FLS but not HFLS invasiveness as determined by transwell assay. Note that CRP-induced RA-FLS proliferation and invasiveness are blocked by neutralizing antibodies against CD32/CD64 (10 mg/ml). Each bar represents the mean ±SEM for at least three independent experiments. *p < 0.05, **p < 0.01 vs. control or as indicated.

As shown in Figures 1A, 2B,C, and Supplementary Figure S1, compared to HFLSs, RA-FLSs were highly activated with proliferative and invasive phenotype and expressed high levels of CRP and its receptors CD32 and CD64 expression, particularly from RA-FLS2. Thus, RA-FLS2 was selected for the further studies *in vitro*. We then investigated the promoter role of CRP in activation of FLSs by stimulating RA-FLSs or HFLS with addition of CRP. As shown in Figure 3, multiplex cytokine assay kits assays showed that addition of CRP dose-dependently upregulated CCL2, CXCL8, IL-6, MMP2, MMP9 in RA-FLS but not in HFLS, although expression of IL-1β and TNFα was not significantly changed (Figure 3A). On the other hand, the production of an anti-inflammatory cytokine IL-10 was also not altered (Figure 3A). Similar results were also detected at the mRNA levels by real- time PCR (Figure 3B). Further studies by the WST-1 and invasiveness assays also revealed that additional of CRP (10 μg/ml) could promote the proliferative and invasive activities by RA-FLS but not by HFLS (Figures 3C,D).

### CRP Signals Through CD32 and CD64 to Differentially Regulate RA-FLS to Produce Pro-inflammatory Cytokines and to Become the Proliferative and Invasive Phenotype *in vitro*

**Figure 4:**
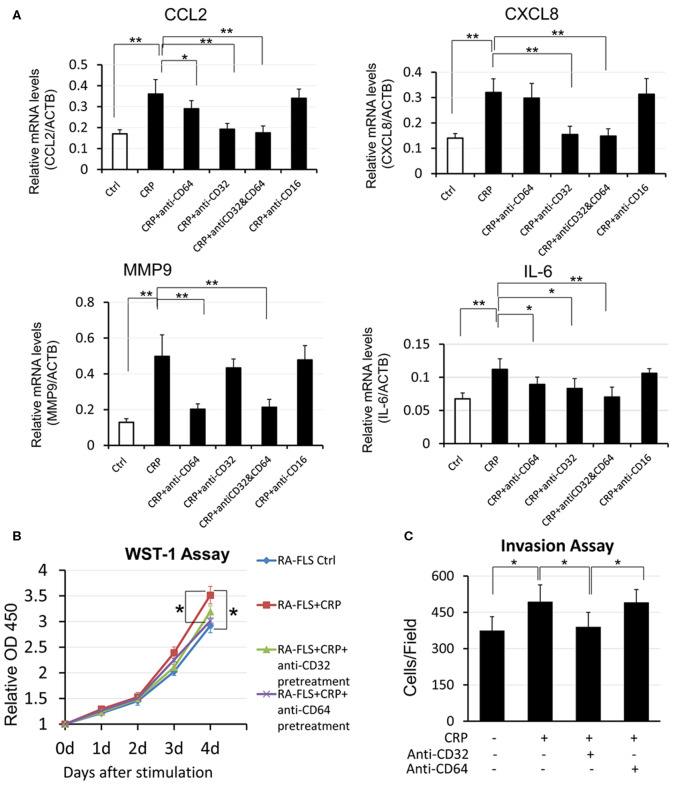
CRP promotes pro-inflammatory responses in RA-FLS differentially via CD32/CD64 signaling. **(A)** Real-time PCR, **(B)** WST-1 proliferative assay, **(C)** transwell invasion assay. Results show that CRP (10 μg/ml)-induced pro-inflammatory cytokines CCL2 andIL-6 are blocked by either neutralizing antibodies to CD32 or CD64 (10 mg/ml), whereas CRP-induced CXCL8 expression, RA-FLS proliferation, and invasion are blocked by neutralizing antibodies to CD32 but not CD64 (10 mg/ml). Interestingly, CRP-induced MMP9 is blocked with antibodies to CD64 but not to CD32. Note that blocking CD16 produces no inhibitory effect on CRP-induced expression of CXCL8, CCL2, MMP9, and IL-6. Each bar represents the mean ± SEM for at least three independent experiments. *p < 0.05, **p < 0.01 as indicated.

It is now well-established that CRP exhibits its pathophysiological effects via its receptors CD32 and CD64. CD32 is a high-affinity receptor FcγRIIa and CD64 is a low- affinity receptor FcγRI (16, 19). We thus blocked CD32 or CD64 or both with neutralizing antibodies to differentially determine the signaling mechanisms of CRP-induced cytokine expression. As shown in Figure 4A, CRP-induced expression of CCL2 and IL-6 was blocked by either neutralizing antibody to CD32 or CD64 or both, suggesting that CRP signals through both CD32/CD64 to induce expression of CCL2 and IL-6. However, CRP- induced expression of CXCL8 was CD32-dependent as it was blunted by the antibody against CD32, whereas CRP-induced MMP9 was blocked by the antibody to CD64, demonstrating that differential signaling mechanisms for CRP in regulating CXCL8 and MMP9 expression in RA-FLSs. A similar mechanism was also found in CRP-induced RA- FLS proliferation and invasiveness in which CD32 but not CD64 was involved (Figures 4B,C). In contrast, blockade of CD16 produced no inhibitory effect on CRP-induced expression of CXCL8, CCL2, MMP9 and IL-6 by RA-FLSs (Figure 4A), suggesting that CRP may not signal through the CD16 to induce joint inflammation *in vitro*.

### CRP Promotes RA-FLS Pro-inflammatory Response Differentially via the CD32/64-p38 and NF-κB-Dependent Mechanisms *in vitro*

**Figure 5:**
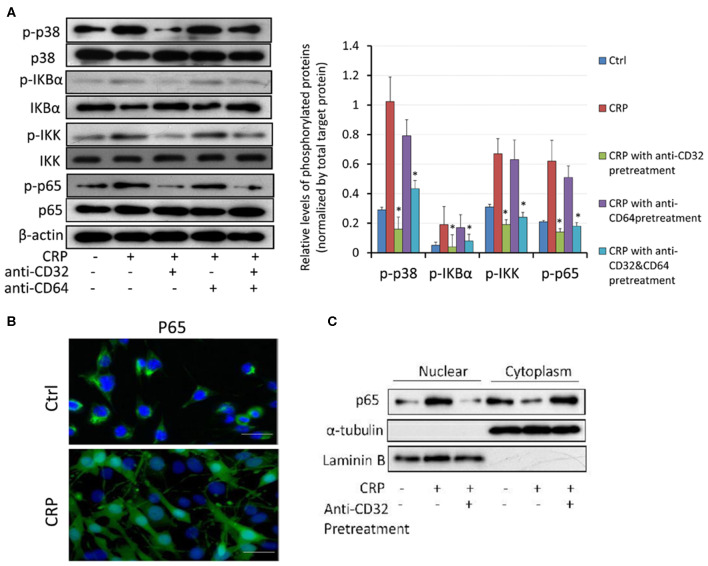
CRP induces activation of p38 and NF-kB/p65 signaling in RA-FLS primarily via the CD32, but not CD64 signaling. **(A)** Western blot analysis and quantitative analysis show that addition of CRP (10 μg/ml) to RA-FLS induces phosphorylation of p38, IκBα, p65 and IKK, which is blocked largely by the neutralizing antibodies to CD32 or CD32+CD64, but not by the anti-CD64 antibody. **(B)** Immunofluorescent staining detects that addition of CRP (10 μg/ml) for 2 h induces nuclear translocation of p65 subunit of NF-κB by RA-FLS. **(C)** Western blot analysis shows that addition of CRP (10 μg/ml) to RA-FLS induces p65 nuclear translocation, which is blocked largely by a neutralizing antibody to CD32. Each bar represents the mean ± SEM for at least three independent experiments. *p < 0.05 compared to CRP stimulation. Scale bar =100 μm.

**Figure 6:**
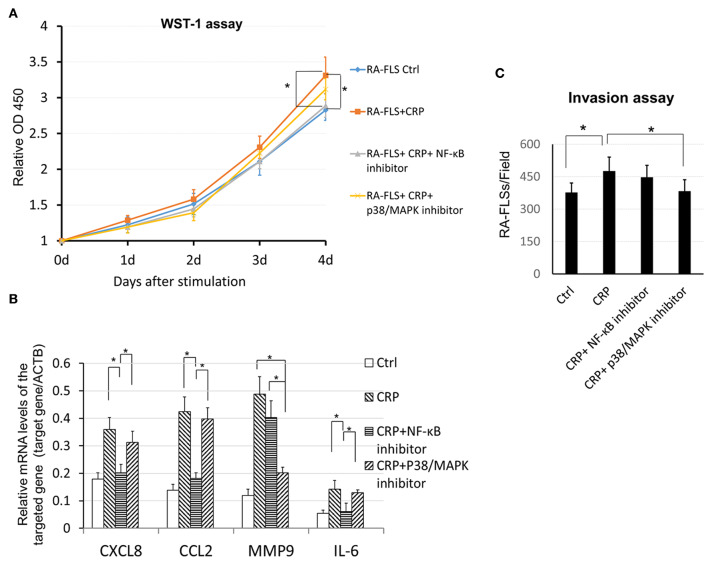
CRP induces proinflammatory cytokine expression, cell proliferation and invasion by RA-FLS differentially via the p38 and NF-κB signaling. **(A)** WST-1 assay shows that pretreatment with a NF-κB inhibitor PDTC (100 μM) but not with a p38 (SB202190, 100μM) significantly blocks CRP (10 μg/ml)-induced RA-FLS proliferation. **(B)** Real-time PCR detects that CRP-induced expression of CXCL8, CCL2, and IL-6 is blocked by a NF-kB inhibitor, whereas, CRP-induced MMP9 expression is inhibited by a p38/MAPK inhibitor. **(C)** Transwell invasion assay reveals that addition of inhibitor to p38 but not NF- κB blocks CRP-induced invasiveness in RA-FLSs. Each bar represents the mean ± SEM for at least three independent experiments. *p < 0.05 as indicated.

It is well-known that NF-κB signaling is a key pathway leading to synovial inflammation in RA and that CRP is capable of activating this pathway in many inflammatory diseases (20, 21). We thus examined if CRP acts via NF-κB signaling to promote activation of RA-FLSs *in vitro*. As shown in Figure 5, addition of CRP (10 μg/ml) was capable of activating p38 and NF-κB signaling as evidenced by increased phosphorylation of p38, IκBα, IKK, p65 (Figure 5A) and by largely promoted p65 nuclear translocation (Figures 5B,C). Importantly, blockade of CRP signaling with neutralizing antibodies to CD32 or CD32/CD64, but not to CD64 alone, largely inactivated CRP-induced phosphorylation of p38, IκBα, IKK, and p65 signaling (Figure 5A), revealing a primary role for CD32-p38/ NF-κB signaling in CRP-mediated activation of RA-FLS. This was further confirmed by the ability of pre-treating RA-FLS with a NF-κB inhibitor, PDTC (100 μmol/L) to inhibit CRP-induced proliferation (Figure 6A) and upregulation of CXCL8, CCL2. and IL-6 but not MMP9 (Figure 6B).

Interestingly, pre-treatment of RA-FLS with a p38/MAPK inhibitor (SB 202190, 100 μmol/L) inhibited CRP-induced RA-FLS invasion (Figure 6C) as well as induction of MMP9 without significant alternation of CXCL8, CCL2, and IL-6 expression (Figure 6B). These data revealed CRP primarily via CD32 to differentially activate p38-dependent MMP9 expression and invasion, and NF-κB-driven CXCL8, CCL2, and IL-6-mediated inflammatory response in RA-FLS.

It has been reported that CRP-induced cytokine expression is also regulated by TWIST transcriptionally in myeloma cells (22). Results shown in Supplementary Figure S2 also clearly demonstrated that addition of CRP was capable of inducing TWIST via the CD32-dependent mechanism as addition of neutralizing antibodies against CD32 but not CD64 blocked CRP- induced TWIST expression by RA-FLSs.

## Discussion

In the present study, we showed that CRP was largely produced in synovial tissues of RA patients. RA-FLS was a major cell type responsible for CRP production in RA patients, accounting for more than 65% of CRP-producing cells as identified by co-expressing CRP and vimentin in the inflamed synovial tissues in patients with RA. The finding of CRP production locally by inflamed synovial tissues may account for increased concentrations of CRP in the joint fluids and elevated serum levels of CRP in patients with active RA as previously reports (23). Furthermore, we also detected that CRP signaling was highly activated with high levels of CD32 and CD64 expression in the synovial tissues of RA patients. Consistent with CRP-expressing cells, FLSs (>65%) was also a major cell-type that highly expressed CD32 and CD64, revealing that CD32/64-expressing FLSs are inflammatory fibroblasts capable of triggering the inflammatory responses in the joint tissues of RA patients in response to CRP. *In vitro* studies confirmed this notion and found that CRP was able to upregulate both CD32 and CD64 and induced FLS proliferation, invasion, and pro-inflammatory expression by increasing production of CCL2, CXCL8, IL-6, MMP2, MMP9 while suppressing an anti-inflammatory cytokine IL-10 expression. Consistent with the known role of CRP in the pathogenesis of other inflammatory diseases such as acute kidney injury (24), obstructive nephropathy (25), atherosclerosis (26), cardiac remodeling (27), hypertension (19), and diabetic kidney disease (28), findings from this also revealed a pathological importance of CRP in synovial inflammation associated with RA.

It is now well-established that CRP exhibits its pathophysiological effects via its receptors CD32 and CD64 (29). In the present study, we found that CRP signaled primarily through CD32, to a less extent of CD64, to differentially regulate joint inflammation. Indeed, CRP-upregulated CCL2 and IL-6 expression was both CD32 /CD64-dependent as neutralizing antibodies to either CD32 or CD64 blocked both CCL2 and IL-6 expression by RA-FLSs. However, blocking CD32 but not CD64 to inhibit CRP-induced FLS proliferation, invasiveness, and proinflammatory cytokine CXCL8 production revealed a major role for CD32 signaling in synovial inflammation, although CRP via CD64, not CD32, to induce MMP9 expression was noticed. Thus, CRP may primarily signal through the high-affinity effect of CD32(FcγRIIa), to a less extent of CD64 (FcγRI), to differentially regulate inflammatory responses as seen in this and other studies (22, 30–38). CRP may also promote synovial inflammations via the CD32-p38 and NF-κB-dependent mechanisms. This was supported by the findings that CRP-induced activation of p38 MAP kinase and NF-κB signaling was blunted by neutralizing antibodies against CD32 but not CD64. Interestingly, CRP-induced MMP9 expression and invasion on RA-FLSs were p38-dependent as addition of a p38 inhibitor (SB202190) but not a NF-κB inhibitor (PDTC) was capable of inhibiting CRP-induced MMP9 expression and cell invasion. In contrast, CRP-induced production of CCL2, CXCL8, and IL-6 was NF-κB-dependent because these inflammatory responses were blocked by an inhibitor to NF-κB but not by a p38 inhibitor. Thus, it is highly possible that high concentration of CRP in synovial fluid in patients with RA may directly bind primarily CD32 to activate p38 MAP kinase and NF-κB signaling in RA-FLSs to differentially regulate synovial inflammation. Blockade of CRP signaling pathway may represent a novel therapy for RA.

It has been reported that CRP-induced cytokine expression is also regulated by TWIST transcriptionally in myeloma cells (22). The present study also demonstrated that addition of CRP was capable of inducing expression of TWIST through the CD32-dependent mechanism, revealing a possible role for TWIST in CRP-induced synovial inflammation, which needs further investigation. In summary, the present study demonstrates that CRP is produced locally in synovial tissues of RA patients, particularly by inflammatory FLSs. Local activation of CRP signaling may contribute significantly to the development of RA. CRP may promote synovial inflammation via mechanism associated with activation of CD32/64- p38 and NF-κB signaling.

## Data Availability Statement

The raw data supporting the conclusions of this article will be made available by the authors, without undue reservation.

## Ethics Statement

The studies involving human participants were reviewed and approved by the Human Research Ethics Committee at the Peking University Shenzhen Hospital. The patients/participants provided their written informed consent to participate in this study.

## Author Contributions

QW and H-YL were involved in the final development of the project and manuscript preparation. ZF, GL, and FZ wrote the manuscript draft. JL, QQ, and JW analyzed the data. ZF, JL, JH, MW, GZ, and YZ performed most of experiments.

## Conflict of Interest

The authors declare that the research was conducted in the absence of any commercial or financial relationships that could be construed as a potential conflict of interest.
