# Supplementary material for: C-Reactive Protein Promotes the Activation of Fibroblast-Like Synoviocytes From Patients With Rheumatoid Arthritis
Source: Front Immunol. 2020 May 20;11:958. doi: 10.3389/fimmu.2020.00958 (PMC7251027; doi:10.3389/fimmu.2020.00958)
Supplement: Supplementary file 1 [file Data_Sheet_1.PDF]

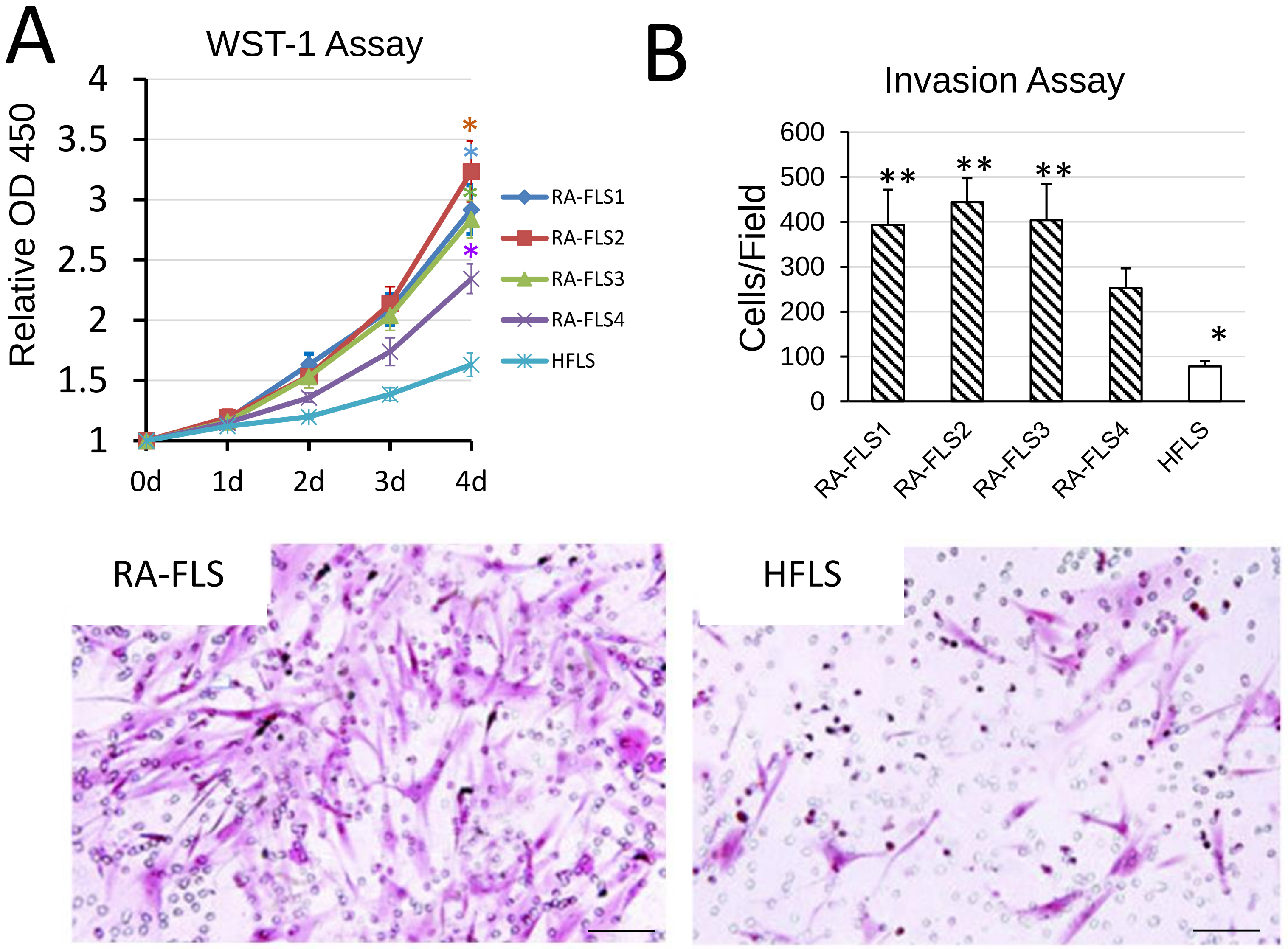

Supplementary Figure S1. Characterizations of RA-FLSs. (A) Proliferative activities of RA-FLS versus HFLS detected by WST-1 assay. (B) Cell invasion assay determined using a transwell assay as described in the Materials and Methods. Microscopic image of migrated cells is shown. Original magnification x 200. Data represent the mean ±SEM for three independent experiments..*p < 0.05 versus HFLS. Scale bar = 50μM

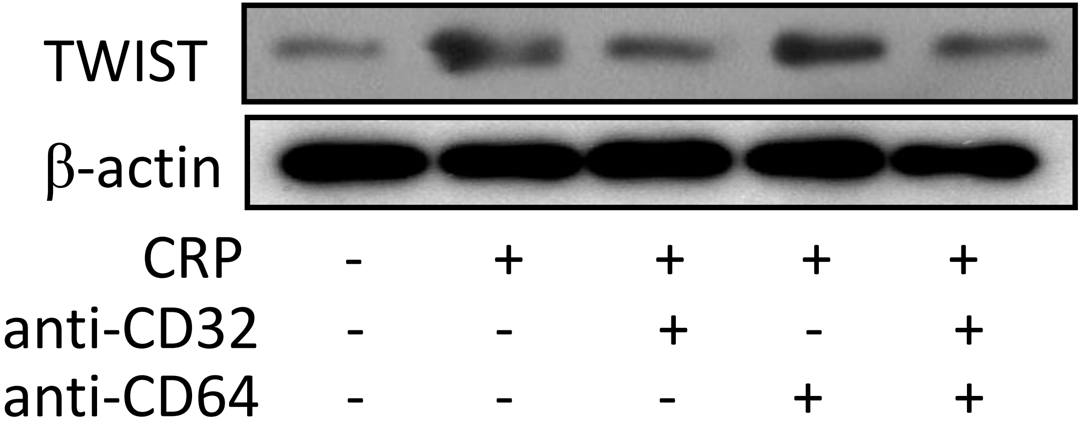

Supplementary Figure S2. CRP induces TWIST expression by RA-FLSs via the CD32-dependent mechanism . Wester blot analysis show that addition of CRP induces TWIST expression by RA-FLSs via the CD32-dependent mechanism as addition of neutralizing antibodies against CD32 but not CD64 block CRP-induced TWIST by RA-FLSs. Data represent 3 independent experiments.
